# Host tree preference and performance of the Eurasian spruce bark beetle ( *Ips typographus* ) on Scots pine

**DOI:** 10.1002/ps.70687

**Published:** 2026-02-22

**Authors:** Jana Gabriele Burchards, Dineshkumar Kandasamy, Martin Nils Andersson

**Affiliations:** ^1^ Department of Biology Lund University Lund Sweden; ^2^ Max Planck Center next Generation Insect Chemical Ecology (nGICE) Lund Sweden

**Keywords:** host choice, monoterpene, pheromone, *Picea abies*, *Pinus sylvestris*, sister broods

## Abstract

**BACKGROUND:**

The Eurasian spruce bark beetle, *Ips typographus*, is one of the most serious pests of Norway spruce (NS, *Picea abies*), with the ability to infest and kill vigorous trees through pheromone‐mediated mass attacks. During outbreaks, *I*. *typographus* has been observed to occasionally attack Scots pine (SP, *Pinus sylvestris*). We investigated in the laboratory whether *I*. *typographus* is attracted to SP bark and logs, which semiochemicals mediate this potential attraction, and how beetle fitness is affected by the host tree species.

**RESULTS:**

*Ips typographus* was attracted to the odors from SP bark plugs in short‐range walking bioassays, and did not prefer the bark odors from NS over SP. In two‐choice bioassays with logs, the beetles also did not discriminate significantly between the tree species. Analysis of NS and SP bark volatiles revealed quantitative differences in abundant monoterpenes and their enantiomers; however, none of the monoterpenes elicited a behavioral response when tested individually. Furthermore, we showed that *I*. *typographus* can reproduce in SP logs but with reduced offspring weight and numbers. Finally, males emitted less of the aggregation pheromone component *cis*‐verbenol when colonizing SP compared with NS.

**CONCLUSION:**

Our results suggest that *I. typographus* is attracted to SP bark odors at short‐range and can reproduce in SP logs under laboratory conditions but with a reduced fitness. The reduced release of *cis*‐verbenol may suggest a reduced likelihood of pheromone‐induced mass attacks on SP. © 2026 The Author(s). *Pest Management Science* published by John Wiley & Sons Ltd on behalf of Society of Chemical Industry.

## Graphical abstract

*Ips typographus* showed lower reproductive output in an occasional host (*Pinus sylvestris*) under laboratory conditions, and did not prefer its main host (*Picea abies*) over *P. sylvestris* in two‐choice bioassays.

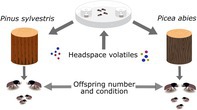

## 1 INTRODUCTION

The Eurasian spruce bark beetle *Ips typographus* L. (Coleoptera: Curculionidae: Scolytinae) typically attacks stressed or weakened trees. However, when the beetle population density is high it infests and kills vigorous trees as well.^1^, ^2^ The main host species of *I*. *typographus* is Norway spruce [NS; *Picea abies* (L.) Karst.], one of the most economical and ecologically important tree species in Europe.^3^ Stress from heat and drought compromises the trees’ chemical defenses and make them more susceptible to bark beetles and pathogens, and these threats are expected to increase because of climate change.^4^, ^5^, ^6^ Moreover, the shallow root system of NS makes them vulnerable to storms, with wind‐felled trees also adding suitable breeding material for *I*. *typographus*. In parallel, higher temperatures reduce the generation time of *I*. *typographus*,^7^ which promotes its population growth. Hence, the combined effects of climate change on *I*. *typographus* and its host tree are expected to result in more frequent bark beetle outbreaks.^8^

Apart from NS, *I*. *typographus* occasionally attacks trees from other genera, including *Pinus*, *Larix*, and *Abies*.^1^ Attacks on Scots pine (SP; *Pinus sylvestris* L.) have been recorded sporadically in the past.^9^, ^10^, ^11^ Such attacks appear to happen more frequently during hot and dry summers when NS trees in the proximity have been infested already (MN Andersson and D Kandasamy, personal observations); hence, it may be possible that *I*. *typographus* becomes an increasing threat to pine trees in a warming climate.

Olfactory cues are important for *I*. *typographus* during the host selection process,^12^, ^13^, ^14^, ^15^, ^16^ although primary attraction to host volatiles alone has not been proven in the field.^17^, ^18^ Once a host tree has been selected by the first male beetles, most likely based on both pre‐ and post‐landing cues,^15^, ^19^, ^20^ they bore into the bark where they emit an aggregation pheromone (2‐methyl‐3‐buten‐2‐ol and (4*S*)‐*cis*‐verbenol), which rapidly attracts large numbers of conspecifics.^21^ Such mass attacks are necessary to overcome the defenses of vigorous trees.

Mating and oviposition occur under the bark and the developing beetles obtain nutrients by feeding on phloem colonized by ectosymbiotic fungi introduced by the parent beetles.^22^, ^23^ The parental beetles may re‐emerge and establish sister broods in new trees before the second generation of beetles emerges. A sister brood can be established by a female without mating a second time if she interrupts oviposition, replenishes her energy reserves through so‐called regeneration feeding, and then resumes oviposition in a new tree.^24^, ^25^, ^26^ In addition to sister broods, female galleries may also be established in new trees in the absence of males after so‐called pre‐emergence mating, where fully developed siblings mate within the natal galleries, after which the females leave to independently initiate a new brood in a new tree.^23^

The main host species NS has evolved defense systems against invaders, consisting of physical and chemical barriers. Resin containing terpenoids provide the first layer of defense and is formed constitutively. The production of resin can also be induced in response to herbivore damage and pathogens.^27^ *Ips typographus* and its symbiotic fungi have evolved mechanisms to metabolize host monoterpenes, which is crucial for the beetles’ reproductive success and development.^23^ For example, the aggregation pheromone component of *I*. *typographus*, (4*S*)*‐cis*‐verbenol, is produced from the host tree monoterpene (−)‐*α*‐pinene.^28^ Hence, for successful colonization of an occasional host tree species, one might expect these trees to provide a similar chemical profile to allow beetle survival and pheromone production.^29^

Host range expansion has occurred in several bark beetle species.^30^, ^31^, ^32^ Novel host tree species might be easier to colonize by the expanding beetle species, because these trees have not evolved under bark beetle selection pressures and may therefore lack proper defense mechanisms.^33^, ^34^ For example, *Dendroctonus ponderosae* overcame a geographic barrier (Rocky Mountains, Canada) and successfully attacked a naive host, *Pinus banksiana* (jack pine). In addition, *D*. *ponderosae* more frequently attacks high elevation *Pinus albicaulis* (whitebark pine),^35^ which is likely due to a warmer climate. Another example of a species that has started to attack naive host trees is the red turpentine beetle (*Dendroctonus valen*s), which is invasive to China.^31^, ^32^ *Ips typographus* has been shown to successfully reproduce in non‐native conifers, including at least six North American spruce species,^36^, ^37^ but with reduced offspring numbers and body weight. These studies have indicated that *I*. *typographus* may well become an economic threat to non‐native spruce species. On the other hand, *I*. *typographus* has been intercepted more than 500 times in North America without establishing reproducing populations and its recent spread in Great Britain has not been dramatic,^38^ suggesting that its ability to successfully invade foreign conifer habitats may be limited.^39^

In its native range, however, it remains unknown why *I*. *typographus* occasionally attacks SP (and other occasional host species) and whether they suffer fitness costs when they do so. SP is the most widely distributed pine species in the world and is of major commercial importance, it is also sympatric with NS in most regions.^40^, ^41^ Compared with NS, SP has a deeper root system and is more drought tolerant and is therefore expected to withstand global warming better.^42^, ^43^ If *I*. *typographus* increasingly attacks SP in a warming climate, Eurasian forestry may suffer serious losses.

To this end, the objectives of the study were to investigate: (i) whether *I*. *typographus* is attracted to SP, (ii) which semiochemicals mediate the potential attraction, and (iii) whether beetle fitness is affected by the host tree species. Considering that natural attacks on SP trees happen occasionally, we hypothesized that *I*. *typographus* would show some attraction to the odors from SP, whereas colonization of SP likely incurs some fitness costs for the beetles.

To test these hypotheses, we used laboratory bioassays in which we compared beetle attraction to NS *versus* SP, using both natural breeding material (logs) and odor blends emanating from bark samples. We further analyzed the content of major conifer monoterpenes, including their enantiomers, in the bark of the two species and tested the behavioral response of *I*. *typographus* to selected individual monoterpenes. To investigate potential fitness consequences, the number of emerging offspring as well as their length and weight were compared between SP‐ and NS‐reared beetles. We further investigated beetle pheromone production and the number, length, and shape of maternal tunnels in NS and SP logs. Our results imply that *I*. *typographus* is attracted to the odor blends of both tree species under laboratory conditions, and the beetles do not show any clear preference for one over the other in shorter distance walking bioassays. However, the beetles produced fewer and lighter offspring, males produced less *cis‐*verbenol, and females constructed fewer maternal tunnels when attacking SP compared with NS logs.

## 2 MATERIALS AND METHODS

### 2.1 Insect rearing and tree material

Laboratory‐reared beetles (from generations 23–26 and 29–35), originating from Asa, Sweden, were used in the experiments. The beetle culture was maintained on natural NS logs, kept at 23 °C, 60% relative humidity (RH), and a 20:4 h light/dark cycle.^44^ Beetles were sex‐separated based on the bristle density on their pronotum and the few individuals with intermediate bristle density that could not be sex‐determined conclusively were excluded from the experiments.^45^ All NS and SP trees used for rearing, experiments, and chemical analyses were harvested between April and October in Nötesjö (55.52°N, 13.38°E) and Vomb (55.66°N, 13.57°E), respectively, both in South Sweden.

### 2.2 Preparation of spruce bark agar

The bark of a freshly cut NS log was removed. The phloem was then peeled, cut into pieces of approximately 1 cm^2^, and stored at −80 °C. A cryo ball mill (Analysette 3 Spartan pulverisette; FRITSCH GmbH, Germany) was used to grind the phloem into a fine powder.^22^ For later use in arena bioassays, 35 mL of agar was poured into Petri dishes (9 cm) under sterile conditions for storage at 4 °C.

### 2.3 Laboratory arena bioassays with bark plugs

**Figure 1:**
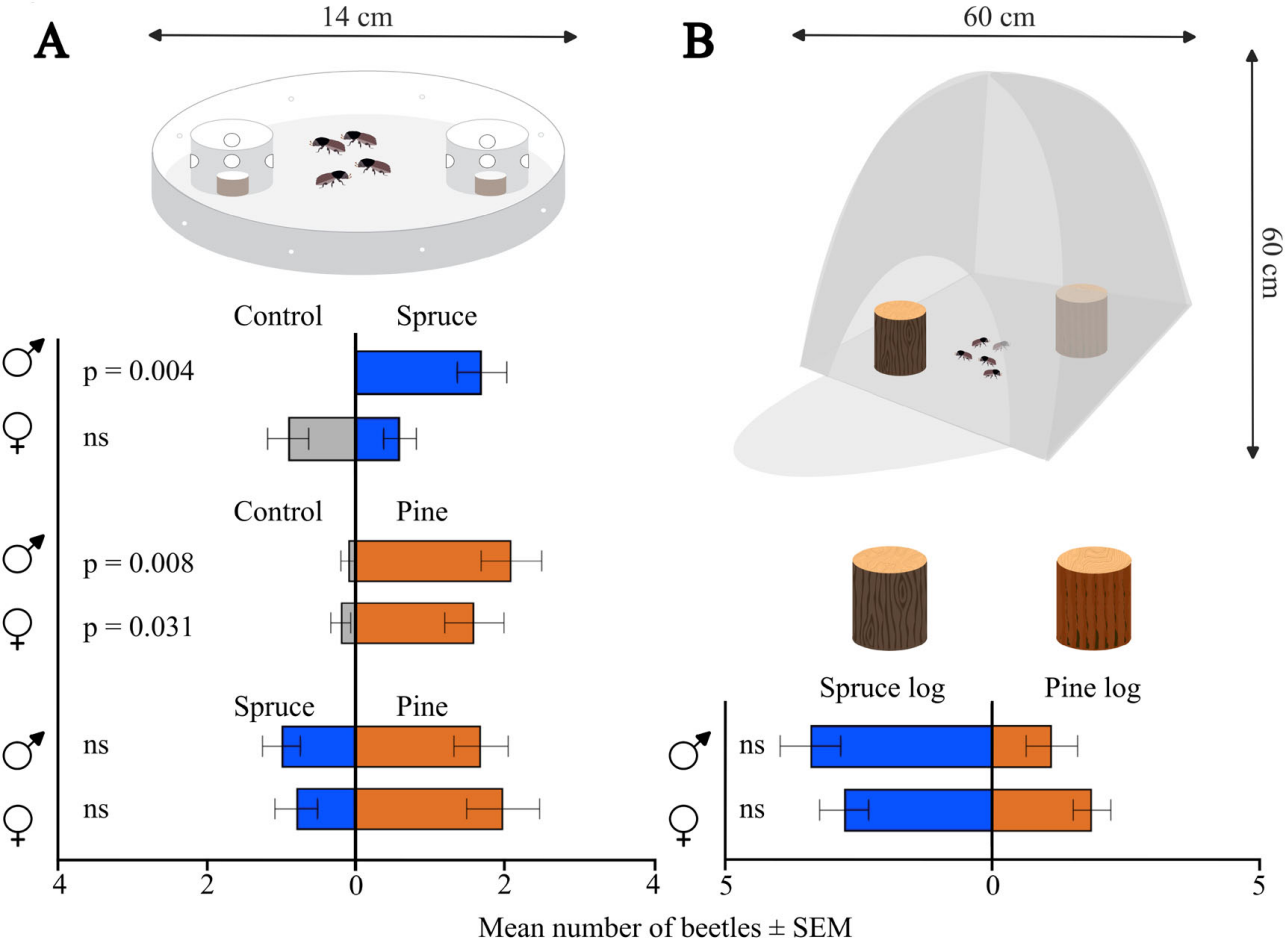
Behavioral laboratory choice assays. (A) Results from short‐range arena bioassays showing attraction of males to Norway spruce (NS) bark and attraction of both sexes to Scots pine (SP) bark when tested against empty control traps. Neither sex discriminated between NS and SP bark (Wilcoxon matched pairs signed rank test, *n* = 10 replicates per sex, each including 4 beetles). (B) Two‐choice assays with NS and SP logs, showing no significant discrimination between tree species (Wilcoxon matched pairs signed rank test, *n* = 8 replicates per sex, each including 5 beetles). ns, non‐significant *P* value.

We performed short‐range arena walking bioassays to assess the behavioral response of *I*. *typographus* to bark samples from NS and SP.^22^ The arenas consisted of glass Petri dishes (14 cm diameter) lined with filter paper. Two plastic cup traps (1.8 cm in diameter) separated by 4.5 cm from each other were placed centrally inside the arena (Fig. 1(A)). The traps had four holes on the sides, big enough to let a beetle enter but trapping them inside after entering. This trap design prevented the beetles from sensing visual and contact cues, while being permeable to volatile cues. Four adult beetles of the same sex were placed in the middle of each arena at the start of each replicate. Males and females were tested separately because *I*. *typographus* often shows sex‐specific responses to odors.^23^, ^46^, ^47^ Each beetle was tested only once. The arena was closed with a lid and the assay was performed in darkness. No external airflow was supplied into the arena during the experiments.

To investigate the behavioral response toward odors from SP and NS bark material, circular bark plugs (1 cm in diameter) were taken from a NS or SP log at a randomly chosen spot. In each arena, one bark plug was put into one of the two traps, whereas one trap remained empty as a control. This assay was performed with NS bark plugs *versus* control, and SP bark plugs *versus* control. To assess whether one of the tree species is preferred by *I*. *typographus*, a two‐choice experiment with an NS bark plug in one trap and an SP bark plug in the other was also performed. Each experiment was replicated ten times per sex, with four beetles in each replicate. Trap catches were counted after 4 h. Beetles that did not make a choice were excluded from the analysis.

### 2.4 Laboratory log selection bioassays

We also performed choice assays at a larger scale with natural logs inside a polyester insect rearing tent (60 × 60 × 60 cm; Bugdorm Insect Rearing Tent, MegaView Science Co., Ltd, Taichung City, Taiwan) under controlled conditions (60% RH, 23 °C), although airflow in the tent was not controlled. One NS log and one SP log (length ~12.5 cm and diameter ~12.5 cm) were placed vertically 30 cm from each other. Five beetles of the same sex were released at the center of the tent, equidistant from the two logs. After 24 h, the number of boring beetles inside the two logs was counted. The experiment was replicated eight times per sex, resulting in a total of 40 beetles tested per sex.

### 2.5 Analysis of headspace bark volatiles and beetle pheromone compounds

To investigate the monoterpene emissions from NS and SP bark, as well as assessing beetle pheromone production, we sampled headspace volatiles of NS and SP logs, sourced from the abovementioned localities in July 2023, May 2023 and September 2024. Three NS and three SP logs of the same diameter (~14 cm) were used. Eight holes were drilled into each log. Four holes were used to measure volatiles in the absence of beetles. The remaining four holes were used to analyze volatiles after beetles had entered (Supporting Information, Fig. S1). Eppendorf tubes (2 mL) were cut open at the bottom, functioning as volatile collection chambers. The tubes were taped to the log, covering the entrance holes. One male beetle was put into each of the four tubes to measure pheromone production and monoterpene emission in response to beetle damage. After 48 h, one female beetle was introduced into each of the four tubes with males. Volatiles from all holes were collected for 30 min every day during 5 days, using solid‐phase microextraction (SPME, 65 μm polydimethylsiloxane/divinylbenzene; Supelco, Bellefonte, PE, USA). The SPME fiber was inserted into the collection chamber through a small hole, using an SPME holder (Supporting Information, Fig. S1). Before collecting volatiles, the fiber was thermally cleaned by inserting it into a gas chromatograph for 5 min at 250 °C (Supporting Information, Method 1).

### 2.6 Chiral analysis of bark extracts

To complement the SPME analysis and to analyze enantiomers of chiral compounds, bark plugs from the same trees used for SPME analysis plus from an additional SP and NS tree (felled in July 2024) were collected and stored at −80 °C. Approximately 15 mg of bark material was extracted for 16 h using 500 μL of *n*‐heptane and analyzed by gas chromatography–mass spectrometry (Supporting Information: Method 2).

### 2.7 Laboratory arena bioassays with single compounds

For bioassays testing individual monoterpenes, we used the short‐range arena setup described in Section 2.3. Each compound was tested against a control. One spruce bark agar (SBA) plug (1 cm in diameter) was placed inside each trap, serving as feeding material. Treatments were randomly assigned to the trap cups. On top of each SBA plug, was placed a round filter paper (Whatman, Maidstone, UK) of the same size. For the control trap, 10 μL of mineral oil (Sigma‐Aldrich, St. Louis, MO, USA) was added to the filter paper. On the treatment trap, 10 μL of the tested compound (one at a time) dissolved in mineral oil was added. This assay was performed with the monoterpenes (−)‐*⍺*‐pinene (>97%, Tokyo Chemical Industry Co., Ltd, Tokyo, Japan), (+)‐*⍺*‐pinene (>97%, Tokyo Chemical Industry), (−)‐*β*‐pinene (>94%, Tokyo Chemical Industry) and racemic 3‐carene (>98%, Sigma‐Aldrich) in different concentrations. (+)‐*⍺*‐Pinene was tested at 1%, 0.1% and 0.01% (v/v) concentrations, whereas (−)‐*⍺*‐pinene, (−)‐*β*‐pinene and (±)‐3‐carene were tested at 10%, 1% and 0.1% concentrations. (+)‐*⍺*‐Pinene was not tested at 10% because this concentration is lethal for the beetles. Each experiment was replicated ten times per sex, with four beetles in each replicate. Trap catches were counted and treated as described in Section 2.3.

### 2.8 Fitness experiment

To investigate the potential fitness consequences for beetles colonizing SP, we compared the reproductive output (number of emerging offspring per log) from NS and SP logs, as well as the weight and length of the emerged offspring. We further compared the number and length of the maternal tunnels, which may correlate to the number of eggs laid by the females. We consider all these measures as proxies for beetle fitness.

To compare offspring numbers and their weight and length, we reared beetles as described above, starting with ten male and ten female beetles in five NS and five SP logs. Logs of the same height and diameter (hence bark area) were used (diameter 12–15 cm; length 25 cm). The numbers of emerged male and female offspring were recorded every day at 10 a.m. for 29 consecutive days. All offspring were weighed and the length of ten offspring from each sex and log were measured (from anterior edge of pronotum to the end of the elytra in dorsal view) using an eyepiece reticle and a stereo‐microscope each day for 13 continuous days. If more than 50 males or females emerged from a log during a single day, 50 random beetles were weighed for representative quantification.

To compare the maternal tunnels between NS and SP, three additional logs of each tree species were placed in rearing boxes, each containing ten male and ten female beetles. After 21 days, the bark was removed, the tunnels were counted, and their length measured from photographs using Fiji ImageJ (v.2.9.0).

### 2.9 Statistical analyses

Data analysis was performed in GraphPad Prism for macOS [v.10.2.3 (347)] and IBM SPSS statistics [v.30.0.0.0 (172)]. Results from walking choice bioassays were analyzed with Wilcoxon matched pairs signed rank test based on the numbers of beetles caught in traps (arena bioassays) or on the number of beetles found inside of the bark of logs (tent assays). In addition, linear regressions were performed based on a response index (RI) against the concentration of tested monoterpenes, to further investigate potential dose‐dependent trends. RI was calculated as: RI = (Number of beetles in treatment trap – Number of beetles in control trap)/Total number of beetles in the arena.

Comparison of the monoterpene content of the SPME volatile collections was performed using mixed effect models, fitted using restricted maximum likelihood (REML) with fixed factors being ‘tree species’, ‘days of repeated measurements’ and the ‘day × tree species’ interaction. ‘Log’ was included as a random factor to account for within‐species interlog‐variation. A Wald *χ* ^2^ tests for fixed effects and a *χ* ^2^ test evaluating the contribution of the random effect variance were performed. Pheromones detected in the volatile collections were compared between NS and SP in the same way as the monoterpene content. To analyze the enantiomeric ratios of monoterpenes in bark samples, the peak area from the identified compounds was normalized to the fresh weight of the analyzed bark plugs. Statistical analysis was performed by taking the average of all replicates and timepoints (*n* = 12) and performing a Mann–Whitney *U*‐test because of unequal variances stemming from zero values for undetected compounds. The cumulative number of offspring emerging from NS and SP logs was analyzed using an unpaired two‐sample *t*‐test. Length and weight data of emerging offspring were analyzed using a factorial General Linear Model (GLM) with the fixed factors ‘sex’ and ‘tree species’, and the ‘sex × tree species’ interaction. Owing to unequal variances (Levene's test), the weight data was log_10_‐transformed prior to statistical analysis. The tunnel length was analyzed using a GLM with ‘tree species’ as a fixed factor. The normality of the weight and length data residuals of beetles and tunnels was validated by manual inspection of Q–Q plots. The number of maternal tunnels in three SP logs was compared with three NP logs using Welch's test. All raw data are available in the Supporting Information raw data file. For clarity, only significant results are reported in the text; all detailed test results can be found in Supporting Information, Tables S1–S6.

## 3 RESULTS

### 3.1 Sex‐dependent behavioral responses to volatiles of NS and SP

Males, but not females, showed attraction toward the volatiles emitted by NS bark plugs, when tested against an empty control trap in short‐range walking arena bioassays (*W* = −45.0, *P* = 0.004; Fig. 1(A)). By contrast, both males and females were attracted to the volatiles of SP bark plugs, when tested against an empty trap (*W*_males_ = −36.0, *P*_males_ = 0.008; *W*_females_ = −32.0, *P*_females_ = 0.031). When given a choice between NS and SP bark plugs, neither of the sexes discriminated significantly between NS or SP bark, although a trend for higher preference for SP was observed in females (Supporting Information, Table S2).

When testing NS against SP in a scaled‐up preference assay with natural conifer logs, neither males nor females showed any significant preference for any conifer species, although a tendency for higher attraction to NS was observed among males (Fig. 1(B); Supporting Information, Table S2).

### 3.2 Different abundances of major monoterpene hydrocarbons in NS and SP, but no short‐range attraction in *Ips typographus*

**Figure 2:**
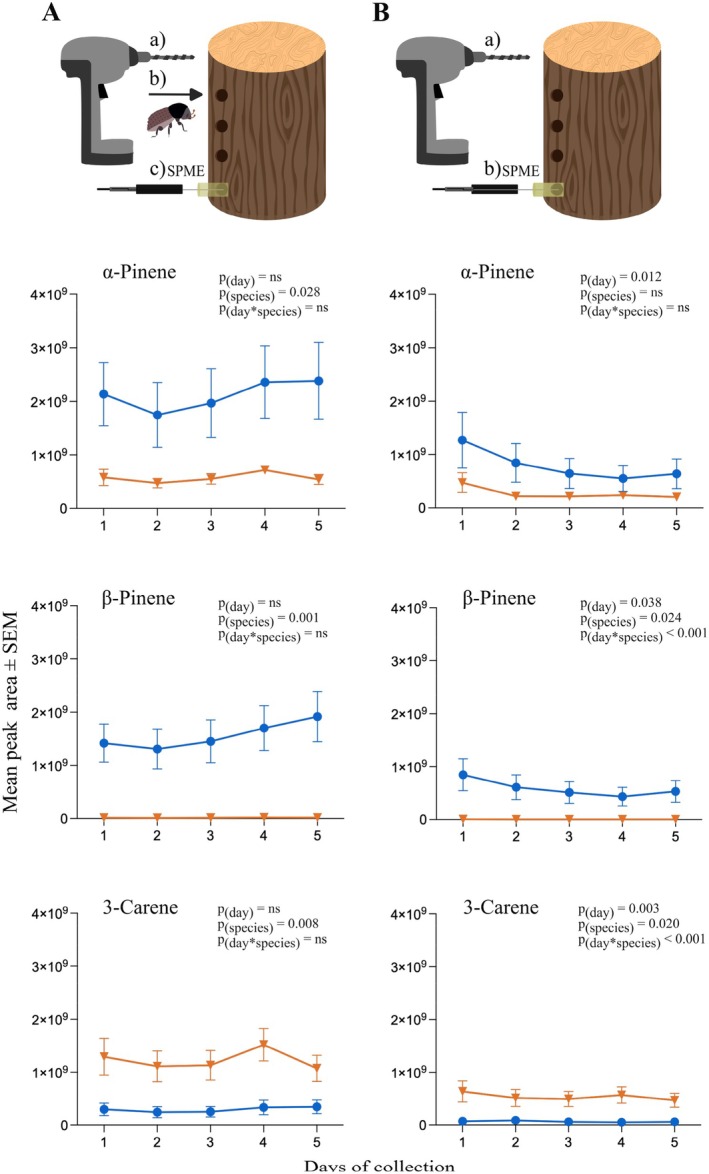
Abundance of the three most abundant monoterpenes in Norway spruce (NS) and Scots pine (SP) over 5 days. (A) Headspace volatile collections from holes with beetles (male present from beginning and female added on after 48 h) show that *⍺*‐pinene and *β*‐pinene were more abundant in NS than in SP. By contrast, 3‐carene was more abundant in SP. (B) Headspace volatile collections from holes without beetles show similar overall patterns as in A, but also variation over time. Statistics based on mixed‐effects model fitted by restricted maximum likelihood (ns, non‐significant *P* value; *n*_total_ = 12). Samples were taken daily for 5 days after drilling holes in the logs.

Headspace volatile collections from drilled holes in NS and SP logs in the absence and presence of *I*. *typographus* were performed to compare monoterpene hydrocarbon emissions. Of ten identified monoterpenes, we focused on the three most abundant across the two tree species (Supporting Information, Fig. S2). We observed differences between NS and SP in the three dominant monoterpenes *α*‐pinene, *β*‐pinene and 3‐carene across the 5 days of sampling. Across the sampling period with a beetle inside, a significantly higher level of *α*‐pinene was found in NS than in SP (*F*_1,22_ = 5.539, *P* = 0.028) (Fig. 2(A); Supporting Information, Table S3). *β*‐Pinene was clearly more abundant in NS than in SP (*F*_1,22_ = 14.24, *P* = 0.001) (Fig. 2(A)), whereas 3‐carene was significantly more abundant in SP across the sampling period (*F*_1,22_ = 8.378, *P* = 0.008) (Fig. 2(A)). The levels of these monoterpenes did not vary significantly over the 5 days of sampling. Similar overall differences in these monoterpenes were observed from the holes without beetles, but at lower levels (Fig. 2(B)). However, because of large variation among the NS samples, the difference in *α*‐pinene between NS and SP in the absence of beetles was not significant (Supporting Information, Table S3), and this compound decreased over time (*F*_0.837,15.91_ = 8.802, *P* = 0.012). Significant ‘day × tree species’ interactions were observed for both *β*‐pinene found only in NS (*F*_4,76_ = 8.942, *P* < 0.001) and 3‐carene (*F*_4,76_ = 5.526, *P* < 0.001), which again was most abundant in SP.

The analysis of monoterpene enantiomers revealed that the SP bark contained (+)‐*⍺*‐pinene, whereas the *cis*‐verbenol precursor (−)‐*⍺*‐pinene was not detected in SP bark (Supporting Information, Fig. S3). By contrast, both enantiomers were detected in NS bark. The level of (+)‐*α*‐pinene did not differ between tree species. (−)‐*β*‐Pinene was detected in NS only, whereas (+)‐*β*‐pinene was not detected in the bark extracts of NS or SP. (+)‐3‐Carene was present in both NS and SP extracts, but was more abundant in SP (*P* < 0.001), akin to the results from the headspace volatile collections. In addition, we observed seasonal variation for the different monoterpenes in both NS and SP (Supporting Information, Fig. S3).

**Figure 3:**
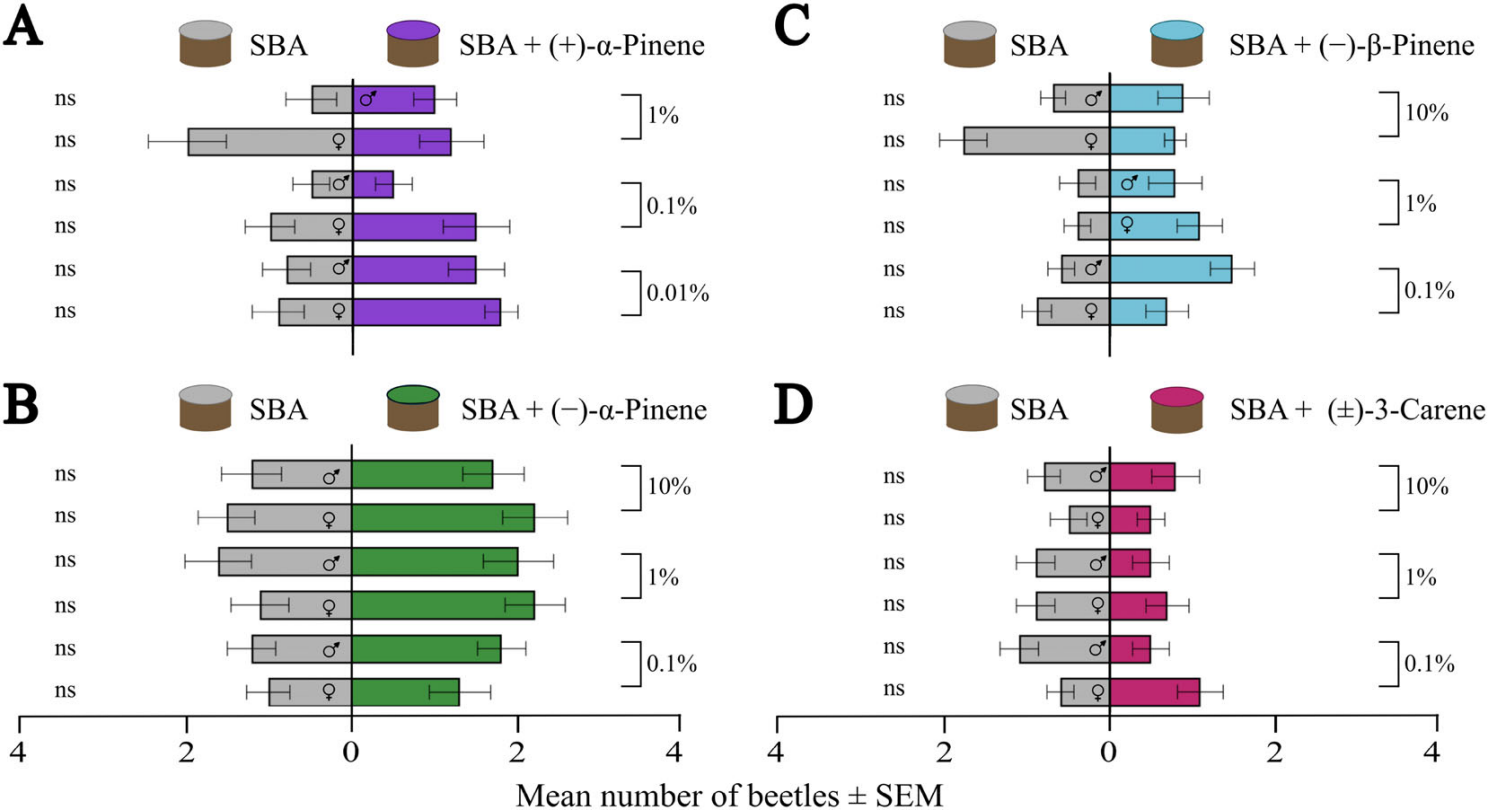
Laboratory walking bioassays with individual monoterpenes. Spruce bark agar (SBA) tested against SBA with (+)‐*⍺*‐pinene (A), (−)‐*⍺*‐pinene (C), (−)‐*β*‐pinene (C) or (±)‐3‐carene (D) added in different concentrations in independent experiments (*n* = 10 replicates, each including 4 beetles). Only beetles that chose a trap were included in the analysis. Numbers of responding beetles are given in the Supporting Information raw data file. Statistics based on Wilcoxon matched pairs signed rank tests. ns, non‐significant P value. See Fig. 1 for the experimental setup.

In laboratory walking choice arena bioassays, *I*. *typographus* did not show any attraction toward the four individual major monoterpenes, tested at several concentrations, nor did they prefer the control trap over any monoterpene (Fig. 3(A)–(D); Supporting Information, Table S4). However, the number of individuals making a choice varied between the different compounds (Supplementary raw data file). When (±)‐3‐carene was presented in different concentrations, only 37% of the beetles responded, whereas the highest response was observed for (−)‐*⍺*‐pinene, with 78% of beetles making a choice. In addition, to investigate potential dose‐dependent effects of the compounds on *I*. *typographus* behavior, response indexes were calculated for each experiment and the indexes were analyzed by linear regressions across the concentration range. Although some overall trends for dose‐dependent effects were observed, none were statistically significant (Supporting Information, Fig. S4; Table S1).

### 3.3 *Ips typographus* pheromone production differs between NS and SP

**Figure 4:**
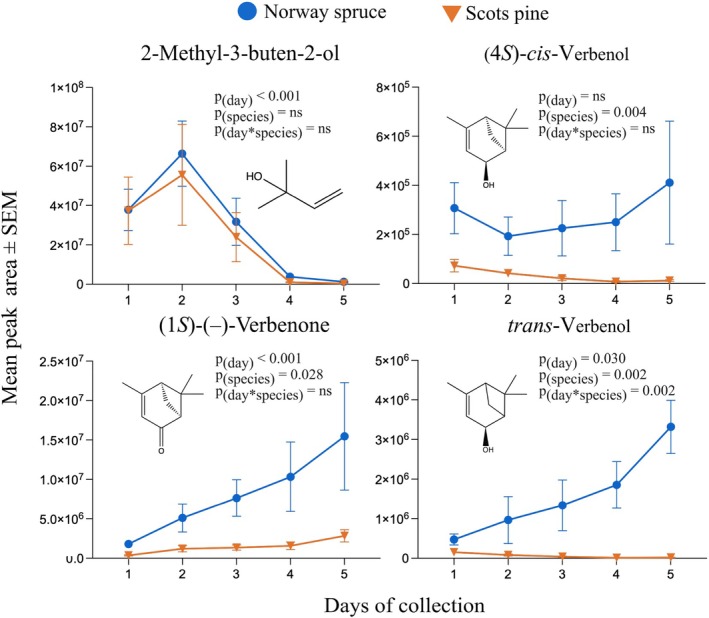
Variation in pheromone production by beetles boring into Norway spruce (NS) and Scots pine (SP) logs. Adult male beetles were present during the whole period of measurements, and one female beetle per male was introduced after day 2 of collections. Statistics based on mixed‐effects model fitted by restricted maximum likelihood (ns, non‐significant *P* value, *n*_total_ = 12).

Adult *I*. *typographus* previously reared on NS were put into predrilled holes in NS or SP logs, and headspace volatiles from these holes were collected over five consecutive days. For the aggregation pheromone component 2‐methyl‐3‐buten‐2‐ol, a significant effect of day of sampling was observed (*F*_1.403,25.95_ = 11.66, *P* < 0.001) (Fig. 4; Supporting Information, Table S5), with peak production at day 2, followed by a steep decline. This was observed for both NS and SP, with no significant difference between tree species, and could partly be due to a mating effect because females were added on day 2. By contrast, significantly more of the other aggregation pheromone component, *cis*‐verbenol, was produced by beetles in NS compared with SP (*F*_1,21_ = 10.58, *P* = 0.004). Likewise, the level of verbenone was significantly higher from NS than from SP (*F*_1,21_ = 5.610, *P* = 0.029) across the sampling period, with emission significantly increasing over time (*F*_4,74_ = 5.377, *P* < 0.001). Also, *trans*‐verbenol levels were higher in NS than in SP (*F*_1,21_ = 12.50, *P* = 0.002) with increased emission over time (*F*_1.838,34_ = 4.00, *P* = 0.030) and a significant ‘day × tree species’ interaction (*F*_4,74_ = 4.866, *P* = 0.002).

### 3.4 Reproduction in SP pine has fitness consequences

**Figure 5:**
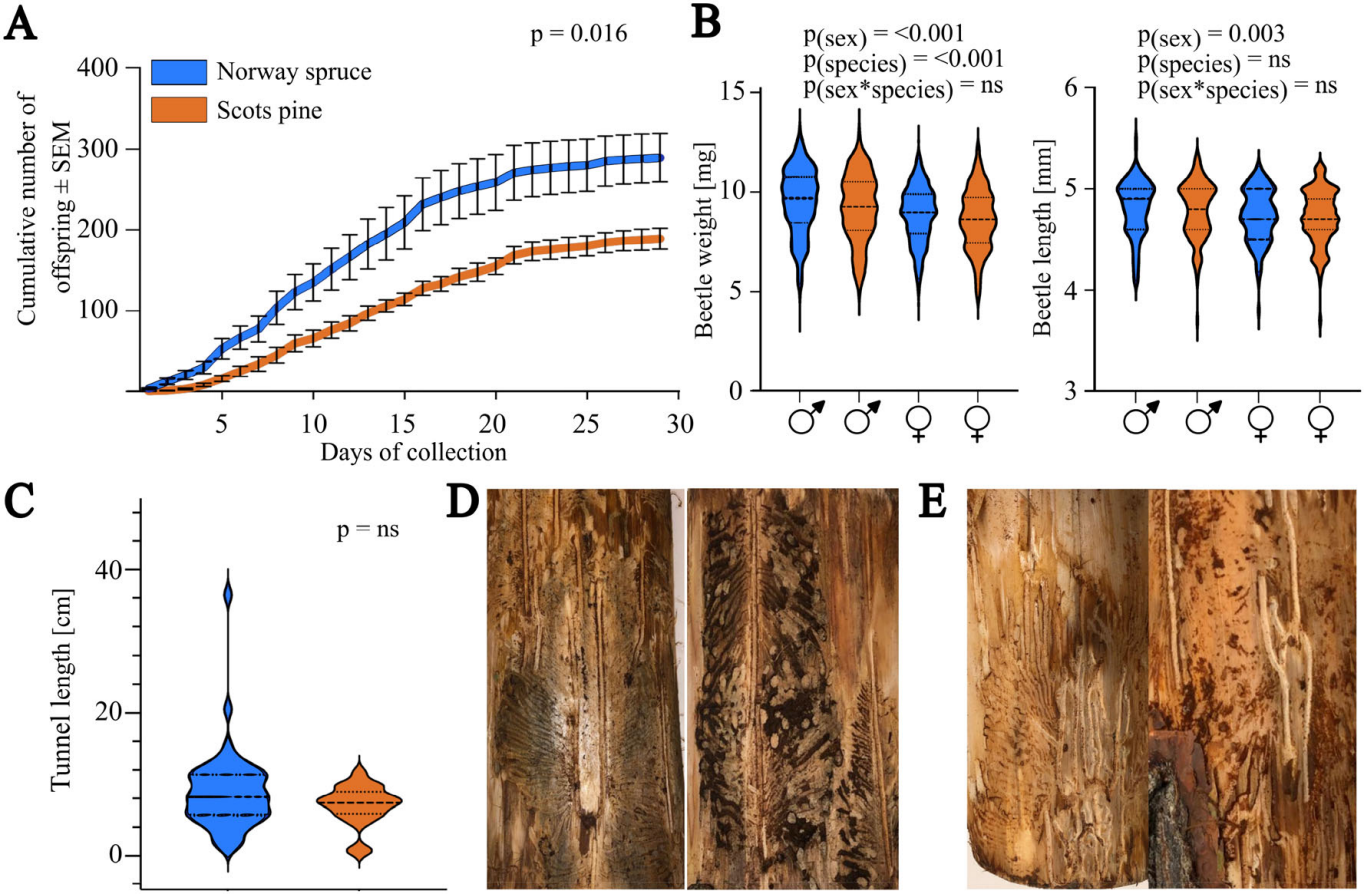
Offspring numbers, weight, length, and maternal tunnels. (A) Average cumulative numbers of emerged offspring over 29 days from Norway spruce (NS) and Scots pine (SP) logs (unpaired *t*‐test; *n*_logs_ = 5 for each tree species). (B) Body weight and length of beetle offspring emerging from NS and SP logs. The dashed line represents the median, and dotted lines the first and third quartiles. (C) Length of maternal tunnels in NS and SP. A total of 45 maternal tunnels were found in NS and 20 in SP (*n* = 3 logs). The dashed line represents the median, and dotted lines the first and third quartiles (statistics based on General Linear Model; ns, non‐significant *P* value). (D) Photographs showing differences in the shape of maternal tunnels as well as fungal staining in NS and (E) SP.

When investigating *I*. *typographus* reproduction in NS and SP logs, we found that significantly fewer offspring emerged from SP than from NS; in total, 1447 offspring emerged from the five NS logs within 29 days and 945 offspring emerged from the same number of SP logs with similar size and bark area (*F*_8_ = 5.167; *P* = 0.016) (Fig. 5(A); Supporting Information, Table S6). The mean sex ratio of the offspring was similar between NS (56.6% males) and SP (54.0% males).

Beetle offspring emerging from NS were significantly heavier than beetles emerging from SP (*F*_1,1965_ = 13.753, *P* < 0.001) (Fig. 5(B)). In addition, emerged males were significantly heavier than females (*F*_1,1965_ = 61.819, *P* < 0.001) (Table 1), irrespective of tree species. Males were also significantly longer than females (F_1,513_ = 9.003, *P* = 0.003); however, beetle length was not affected by the tree species (Supporting Information, Table S6).

**Table 1:** Weight and length of *Ips typographus* offspring emerged from Norway spruce and Scots pine

	Sex	Mean weight ± SD (mg)	*n*	Mean length ± SD (mm)	*n*
Norway spruce	Male	9.53 ± 1.68	645	4.81 ± 0.27	130
	Female	8.86 ± 1.43	553	4.73 ± 0.26	129
Scots pine	Male	9.22 ± 1.70	416	4.79 ± 0.28	129
	Female	8.62 ± 1.53	355	4.72 ± 0.27	129

The total number of maternal tunnels was higher in NS (*t* = 9.449, df = 3, *P* < 0.001), whereas no significant difference in tunnel length was observed between tree species (Fig. 5(C); Supporting Information, Table S6). On average, 7 maternal tunnels were observed in the SP logs with an average length of 7.08 cm compared with 15 tunnels with an average length of 8.94 cm in NS. The shape of the maternal tunnels in SP differed from the typical straight longitudinal tunnels in NS. The tunnels in SP were often winding, going back and forth along the log. In addition, the tunnels in SP generally showed fainter staining from symbiotic fungi compared with tunnels in NS (Fig. 5(D)–(E)).

## 4 DISCUSSION

We conducted this study after reoccurring *I*. *typographus* attacks were observed on SP during recent hot and dry summers. We showed that *I*. *typographus* can reproduce in SP logs under laboratory conditions and is attracted to SP logs and odors emanating from SP bark in small‐scale choice bioassays. In fact, our results suggest that *I*. *typographus* does not prefer its main host species NS over SP at the tested scale. However, they suffer from reduced pheromone production, and produced fewer and lighter offspring when colonizing SP. These findings suggest that the attraction to SP is non‐adaptive, at least when the beetles have the opportunity to colonize NS at low densities and minimal competition.

### 4.1 Males follow host cues, whereas mated females might drive colonization of SP

The males of *I*. *typographus* select which host tree to colonize and subsequently attract conspecifics using an aggregation pheromone.^21^ The fact that only male beetles were attracted to bark plugs from NS highlights their role as pioneers and underscores the importance of the pheromone for NS colonization by females. Interestingly, both sexes showed a surprisingly strong attraction toward bark plugs from SP, and neither sex preferred NS over SP bark in the arena experiments, suggesting that the olfactory cues emitted by both tree species are similarly attractive. These results contrast with those of a previous study in which essential oils extracted from SP twigs and needles showed a repellent effect in an olfactometer bioassay. This difference could potentially be explained by the different plant tissues used, releasing different volatile blends.^48^, ^49^ However, in our scaled‐up choice assays using logs, a non‐significant trend emerged, with both sexes, especially males, showing a slight preference for the main host, NS. It is likely that the larger‐scale setup in which multiple cues, including olfactory, visual, tactile and gustatory, were available, influenced the host selection behavior. In the natural environment, the relative importance of pre‐landing *versus* post‐landing cues for host selection by *I*. *typographus* still remains unknown,^15^, ^19^ and it has been shown that both sexes generally spend a relatively long time investigating the bark after landing.^20^

In the natural environment, it is possible that female beetles can establish galleries in SP in the absence of pheromone‐releasing males, as they do in NS.^24^, ^26^, ^50^ Although the mating status of our experimental females was unknown, our finding that they were attracted to SP and not to NS in the absence of males aligns with the hypothesis of gallery establishment in SP without involvement of male‐produced pheromones. Such establishment could be a spillover effect, in which high population density within the natal host species drives mated females to disperse and colonize new hosts, even to suboptimal species that reduce their fitness.^51^

### 4.2 Quantitative differences in major NS and SP monoterpenes

Our headspace volatile analysis of bark with and without boring beetles demonstrated that (−)‐*α*‐pinene and (−)‐*β*‐pinene, key monoterpenes associated with conifer defense, were present in NS but not detected in SP, whereas (+)‐3‐carene was more abundant in SP. Different ‘chemotypes’ of SP exist in nature, with some individuals having high levels of (+)‐3‐carene (as in the current study) whereas others are richer in *α*‐pinene.^52^, ^53^ In contrast to the 3‐carene chemotype, the *α*‐pinene chemotype contains (−)‐*α*‐pinene, but at lower levels than (+)‐*α*‐pinene.^43^ Like other studies showing no primary attraction to individual monoterpenes, *I*. *typographus* did not exhibit any attraction (or aversion) to any of the monoterpenes tested in our walking bioassays, suggesting that these enantiomers alone are not used or are insufficient for host discrimination at this small scale. This aligns with the concept that bark beetles, like many other insect species, may rely on complex volatile blends rather than single compounds to assess host suitability,^15^, ^54^ or compounds that were not included in our experiments.

Although our bioassays did not reveal any significant behavioral response to 3‐carene, it is notable that only 37% of the beetles made a choice in the presence of this compound; the remaining individuals appeared immobilized. This may reflect a toxicity effect, with 3‐carene being the third most toxic conifer monoterpene to *I*. *typographus*.^55^ However, it remains unknown whether the immobilization was a result of direct toxicological effects or simply due to reduced motivation to move. Furthermore, the presence of high levels of (+)‐3‐carene in SP bark could reduce female fecundity^56^ and may be toxic to larvae, as has been shown in adults,^55^ thereby reducing fitness.

### 4.3 Emission of an aggregation pheromone component is reduced during attacks on SP

We demonstrated that the host tree monoterpene composition affects pheromone emission in *I*. *typographus* differently depending on the biosynthetic pathway of the pheromone. The *de novo* produced aggregation pheromone 2‐methyl‐3‐buten‐2‐ol was emitted at similar levels by beetles boring into NS and SP, which is expected because its biosynthesis is independent of host‐derived precursors.^57^, ^58^ By contrast, the release of *cis*‐verbenol and its oxidized derivate, verbenone, was significantly reduced from holes with beetles boring into SP compared with NS. This reduction is likely due to the absence of (−)‐*α*‐pinene in the analyzed SP bark. Because *cis‐*verbenol is synthesized from (−)‐*α*‐pinene, this supports previous observations of a strong link between monoterpene precursor availability and pheromone output.^59^ The observed low emission of *cis*‐verbenol in SP logs could arise from the alternative biosynthetic pathway that does not require immediate contact with the precursor (−)‐*α*‐pinene. (−)‐*α*‐Pinene can be converted to verbenyl esters, which are stored in the fat bodies of males that have fed on NS before dispersal and these esters can then be converted into *cis*‐verbenol in a new host, allowing a low pheromone release upon initial colonization in SP.^57^ However, without provision of (−)‐*α*‐pinene from SP, sustained *cis*‐verbenol production is unlikely, as suggested by our data. Because both *cis*‐verbenol and 2‐methyl‐3‐buten‐2‐ol need to be present to trigger attraction in the field, the compromised *cis*‐verbenol production is likely to result in reduced chances of mass attack initiation and/or a lower colonization density of *I*. *typographus* on SP; however, this hypothesis remains to be tested. In addition, *I*. *typographus* pheromone production should be investigated from SP chemotypes that contain (−)‐*α*‐pinene,^52^ because the production of *cis*‐verbenol is likely to be higher in such trees. The non‐detectable levels of (−)‐*α*‐pinene in SP may have broader implications for host attractiveness, because high concentrations of this compound can synergize attraction to the aggregation pheromone.^18^, ^60^ Collectively, our findings highlight the importance of host‐derived chemical precursors in mediating pheromone‐driven behaviors and suggest potential challenges for successful male‐induced mass attacks on SP.

### 4.4 *Ips typographus* suffers from reduced fitness when reproducing in SP

Our breeding experiments demonstrated that although *I*. *typographus* can reproduce in cut SP logs, the number of emerging offspring is significantly reduced compared with reproduction in NS, similar to studies conducted on lodgepole pine (*Pinus contorta*).^11^, ^61^ These findings suggest that *I*. *typographus* may have limited ability to invade and establish populations in non‐*Picea* tree species; indeed, a recent study suggested that their breeding success in cut Sitka spruce (*Picea sitchensis*) material was similar to that in NS,^33^ although a previous study showed reduced total offspring production in this tree species.^62^ In the current study, the lower number of offspring emerging from SP could result from reduced oviposition by females, potentially explained by the unusual maternal tunnel shape in SP and the general lower number of maternal tunnels. Another possibility is decreased larval survival, for example, due to suboptimal nutritional conditions or defense chemicals, such as phenolics and terpenes. Indeed, offspring from SP showed significantly lower body weights, and visual observations indicated premature emergence in some individuals—both signs of nutritional stress and accelerated development under unfavorable conditions.^63^ Interestingly, beetle body length was not affected by the host tree species, suggesting that body mass, which is more directly linked to energy reserves and fitness, is a better indicator of host quality.

A previous study observed similar occurrences of ophiostomatoid fungi in *I*. *typographus* galleries from NS and SP bark.^9^ However, we observed that our laboratory‐reared beetles’ (for which *Ophiostoma bicolor* and *Grosmannia penicillata* are the dominant symbionts) galleries in SP had reduced staining associated with the growth of such fungi. Fungal growth might be inhibited by defense chemicals, such as phenolics in SP that are different from those in NS,^64^, ^65^ and it is possible that the inability of symbiotic fungi to grow well in SP may result in nutritional deficiencies for the beetles.^22^, ^23^ The observed reduced offspring weight and poor establishment of fungal symbionts indicate that it may be challenging for *I*. *typographus* to maintain their populations in SP across multiple beetle generations. Other bark beetle species have also been observed to attack suboptimal host species, including non‐natal ones. For example, *Polygraphus proximus* preferred an exotic *Abies* species despite low colonization success and displayed a lower preference for a host species where it had high colonization success rate.^66^ The proximate and ultimate drivers for such preference‐colonization mismatches in bark beetles remain to be elucidated.

## 5 CONCLUSIONS

Our findings demonstrate that *I*. *typographus* is attracted to the odors of SP and is capable of reproducing in logs from these trees, but they suffer fitness costs when doing so. The observed reduced fitness, compromised production of *cis*‐verbenol, and the seemingly poor establishment of symbiotic fungi suggest that sustained population persistence of *I*. *typographus* in SP may be unlikely. Whereas our study focused on laboratory bioassays, attacking living, more well‐defended, SP trees in the natural habitat could have even larger fitness consequences than those observed in the current study.^67^ In addition, *I*. *typographus* may face interspecific competition in SP with established SP specialists, including species from for example, the *Tomicus* and *Ips* genera. Hence, SP does not offer an unoccupied niche for *I*. *typographus*. Nonetheless, this species’ ability to successfully attack and kill SP, possibly through female‐initiated establishments, poses a potential threat to forestry during high beetle population densities. Future studies should further investigate why *I*. *typographus* suffers from reduced fitness in pine and especially investigate its attacks and success on standing trees in the natural environment.

## CONFLICT OF INTEREST

The authors declare no conflict of interest.

## Supporting information


**Data S1.** Supporting Information.

## Data Availability

The data that supports the findings of this study are available in the supplementary material of this article.
