# Supplementary material for: Host tree preference and performance of the Eurasian spruce bark beetle ( Ips typographus ) on Scots pine
Source: Pest Manag Sci. 2026 Feb 22;82(6):5850–60. doi: 10.1002/ps.70687 (PMC13158442; doi:10.1002/ps.70687)
Supplement: Supplementary file 1 — Data S1. Supporting Information. [file PS-82-5850-s001.docx]

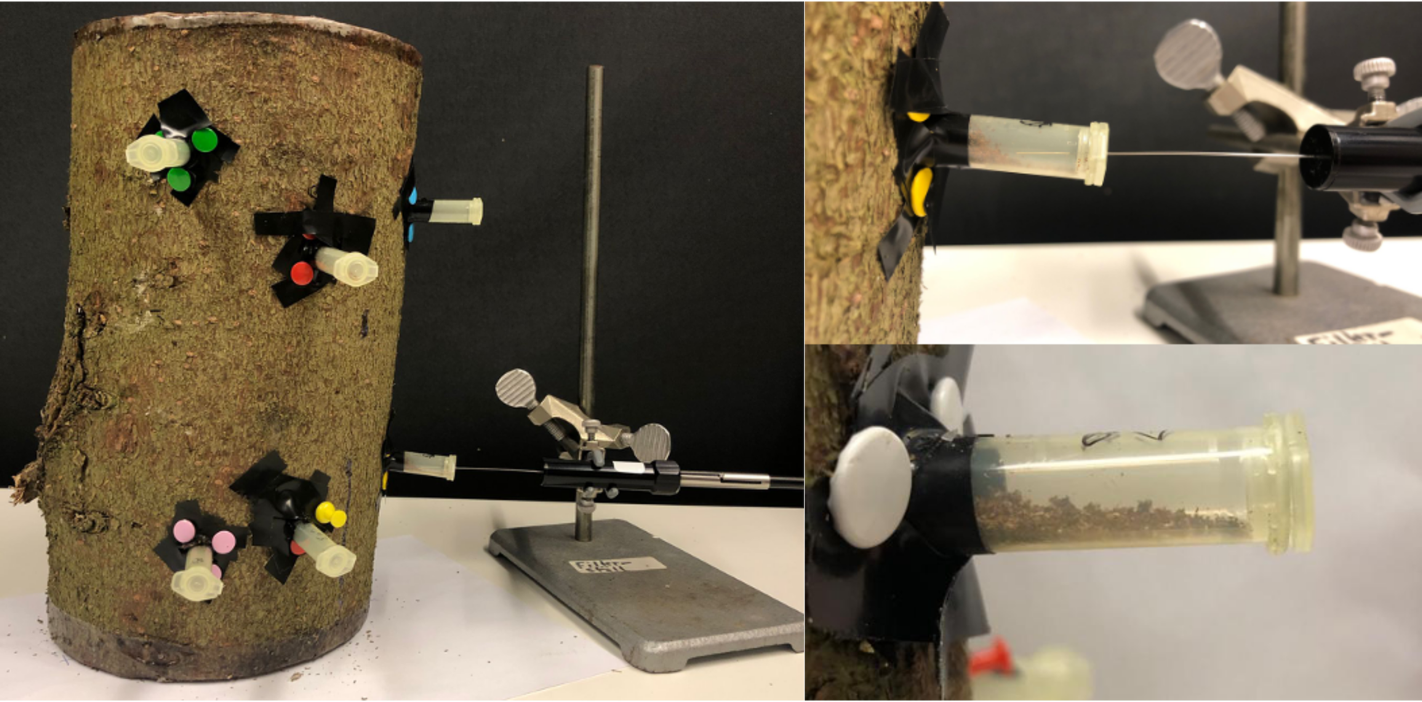


**Figure S1**: Left: Setup for headspace volatile collections. Right: SPME fiber inserted into collection chamber that is constructed fusing an Eppendorf tube (upper photo) and collection chamber with frass (lower photo).


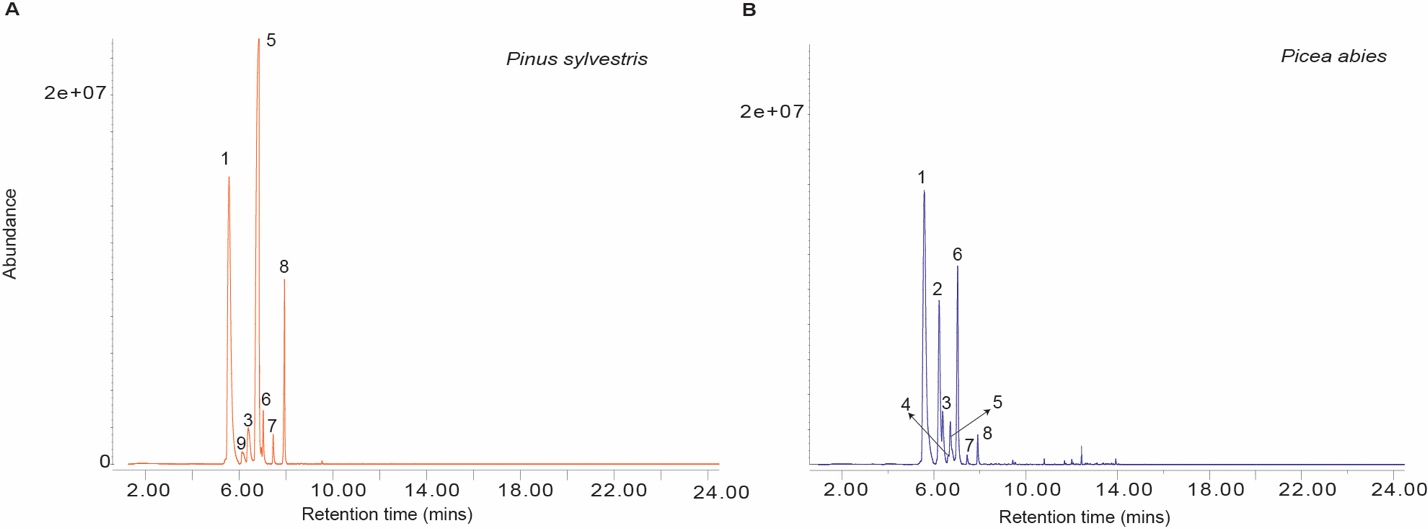


**Figure S2**: Representative chromatograms from headspace volatile collections of Pinus sylvestris (A) and Picea abies (B) using gas chromatography-Mass Spectrometry (GC-MS), collected using the set-up described in Figure S1. Main peak numbers: 1. α-Pinene 2. β-Pinene. 3. Myrcene. 4. α-Phellandrane. 5. 3-Carene. 6. β-Phellandrane/Limonene. 7. γ-Terpinene. 8. Terpinolene. 9. α-Thujene.


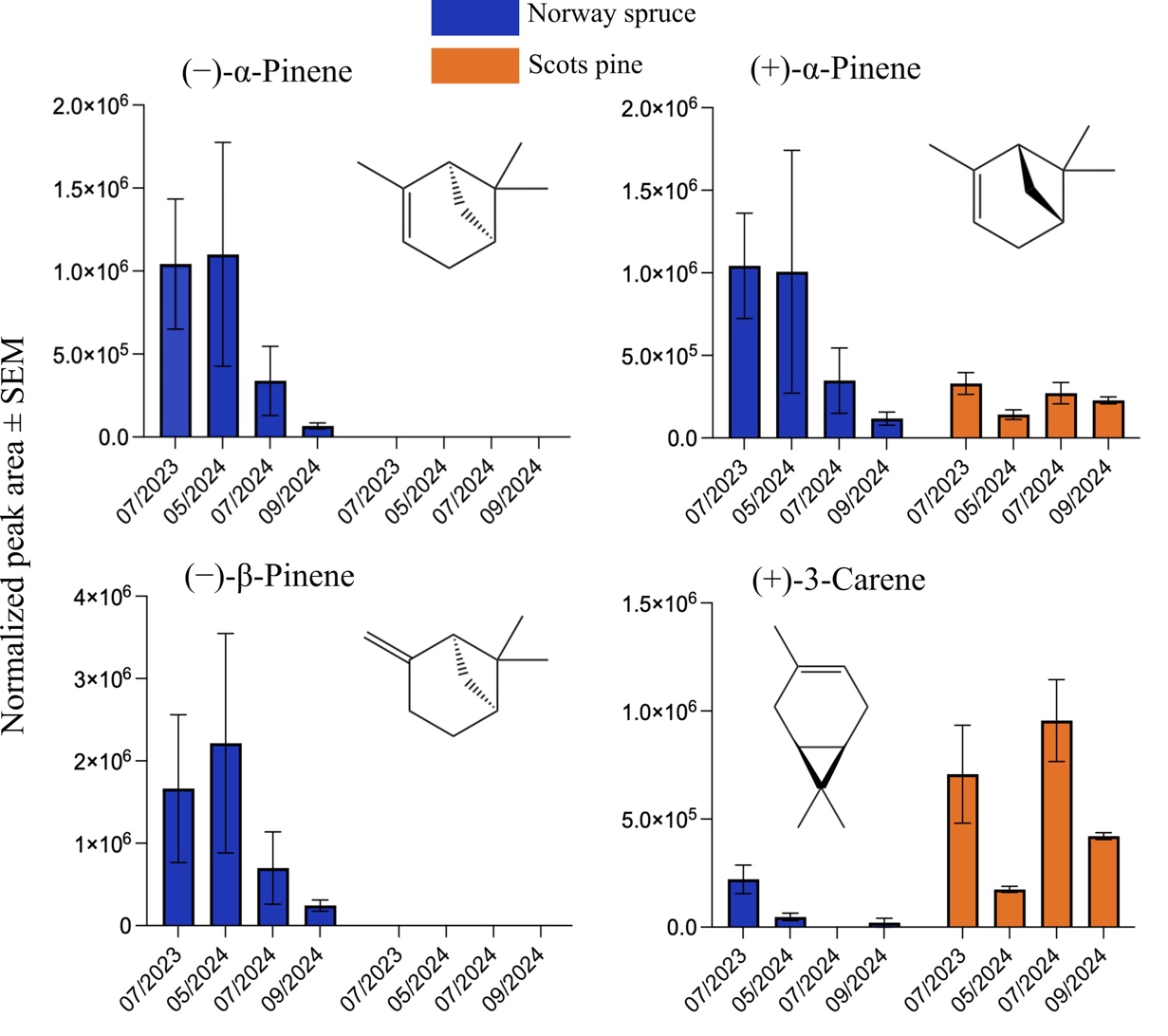


**Figure S3:** Normalized peak area of the dominant monoterpenes from the bark extracts of Scots pine and Norway spruce, sampled during different years and months (n = 4 per sampling date).

**
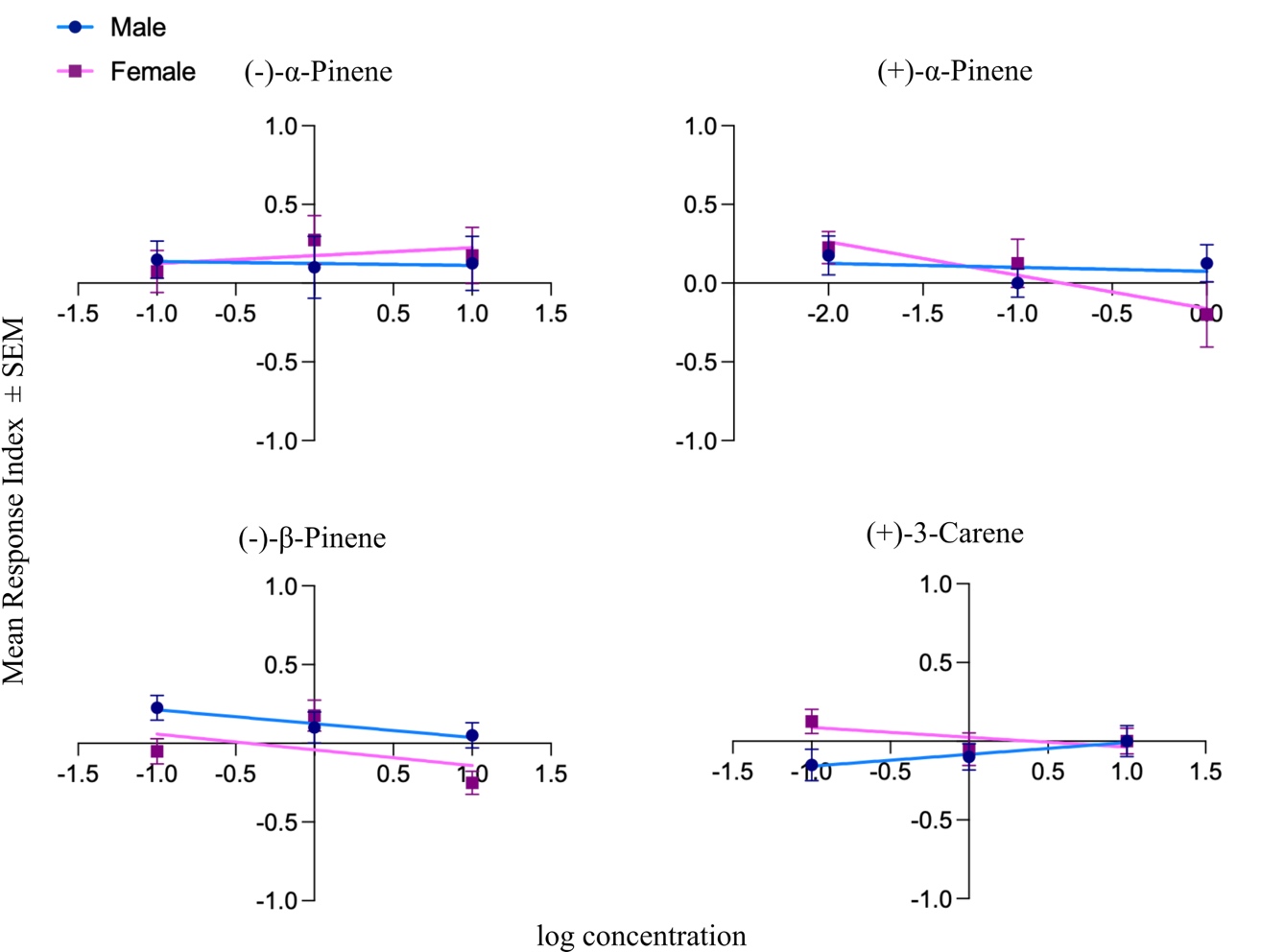
Figure S4:** Linear regression performed with the response index (RI) against the log concentration of tested monoterpenes. A positive RI means more beetles chose the compound over the control and vice versa. Lines are fitted based on raw data, whereas mean values ± SEM are plotted for legibility (n = 10 replicates per sex, each including 4 beetles)

**Table S1**: R^2^, p-values and equations for the linear regressions performed for the data presented in Figure S4.

| **Monoterpene** | **Sex** | **R^2^** | **p-value** | **Equation** |
| --- | --- | --- | --- | --- |
| (-)-α*-*Pinene | Male | 0.0004184 | 0.9146 | Y = -0.01250*X + 0.1250 |
|  | Female | 0.007253 | 0.6545 | Y = 0.05000*X + 0.1750 |
| (+)-α*-*Pinene | Male | 0.003497 | 0.7563 | Y = -0.02500*X + 0.07500 |
|  | Female | 0.1140 | 0.0681 | Y = -0.2125*X - 0.1625 |
| (-)-β*-*Pinene | Male | 0.06901 | 0.1608 | Y = -0.08750*X + 0.1250 |
|  | Female | 0.06931 | 0.1598 | Y = -0.1000*X - 0.04167 |
| Racemic 3-carene | Male | 0.04426 | 0.2645 | Y = 0.07500*X - 0.08333 |
|  | Female | 0.03406 | 0.3289 | Y = -0.06250*X + 0.02500 |

**Table S2**: Statistics for results presented in Figure 1. Wilcoxon matched pairs signed rank test was performed. Significant p-values (p < 0.05) indicated in bold.

| **Figure** | **Experiment** | **Sex** | **Sum of signed ranks (W)** | **p-value** |
| --- | --- | --- | --- | --- |
| 1A | Spruce vs Ctrl | Male | -45.00 | **0.0039** |
|  |  | Female | 10.00 | 0.4375 |
|  | Pine vs Ctrl | Male | -36.00 | **0.0078** |
|  |  | Female | -32.00 | **0.0312** |
|  | Spruce vs Pine | Male | 15.00 | 0.3750 |
|  |  | Female | 20.00 | 0.1719 |
| 1B | Spruce vs Pine | Male | -14.00 | 0.2344 |
|  |  | Female | -21.00 | 0.0938 |

**Table S3**: Statistics for results presented in Figure 2. Mixed-effects model fitted by REML was performed. Significant p-values (p < 0.05) indicated in bold.

| **Figure 2A** | | | |
| --- | --- | --- | --- |
|  | **α*-*Pinene** | **β*-*Pinene** | **3-Carene** |
| p (day) | 0.2080 | 0.3613 | 0.1835 |
| F (dfn, dfd) | F (2.157, 42.59) = 1.622 | F (1.593, 31.45) = 1.005 | F (1.166, 23.03) = 1.881 |
| p (species) | **0.0279** | **0.0010** | **0.0084** |
| F (dfn, dfd) | F (1, 22) = 5.539 | F (1, 22) = 14.24 | F (1, 22) = 8.378 |
| p (day*species) | 0.4361 | 0.4419 | 0.5701 |
| F (dfn, dfd) | F (4, 79) = 0.9566 | F (4, 79) = 0.9461 | F (4, 79) = 0.7361 |
| **Figure 2B** | | | |
|  | **α*-*Pinene** | **β*-*Pinene** | **3-Carene** |
| p (day) | **0.0118** | **0.0382** | **0.0026** |
| F (dfn, dfd) | F (0.8372, 15.91) = 8.802 | F (0.3219, 6.116) = 9.105 | F (1.455, 27.64) = 8.827 |
| p (species) | 0.1599 | **0.0239** | **0.0196** |
| F (dfn, dfd) | F (1, 22) = 2.116 | F (1, 22) = 5.887 | F (1, 22) = 6.339 |
| p (day*species) | 0.1541 | **<0.0001** | **0.0006** |
| F (dfn, dfd) | F (4, 76) = 1.721 | F (4, 76) = 8.942 | F (4, 76) = 5.526 |

**Table S4**: Statistics from Wilcoxon matched pairs signed rank test shown in Figure 3.

| **Figure** | **Experiment** | **Conc.** | **Sex** | **Sum of signed ranks (W)** | **p-value** |
| --- | --- | --- | --- | --- | --- |
| 3A | SBA vs.  SBA + (+)-α*-*Pinene | 1% | Male | -9.000 | 0.4375 |
|  |  |  | Female | 16.00 | 0.4062 |
|  |  | 0.1% | Male | 0.000 | >0.9999 |
|  |  |  | Female | -12.00 | 0.4297 |
|  |  | 0.01% | Male | -21.00 | 0.1484 |
|  |  |  | Female | -24.00 | 0.1016 |
| 3B | SBA vs.  SBA + (-)-α*-*Pinene | 10% | Male | -10.00 | 0.5703 |
|  |  |  | Female | -15.00 | 0.4141 |
|  |  | 1% | Male | -7.000 | 0.6953 |
|  |  |  | Female | -15.00 | 0.1562 |
|  |  | 0.1% | Male | -21.00 | 0.2773 |
|  |  |  | Female | -5.000 | 0.8359 |
| 3C | SBA vs.  SBA + (-)*-*β*-*Pinene | 10% | Male | -7.000 | 0.7656 |
|  |  |  | Female | 38.00 | 0.0273* |
|  |  | 1% | Male | -11.00 | 0.4688 |
|  |  |  | Female | -20.00 | 0.1094 |
|  |  | 0.1% | Male | -30.00 | 0.0469* |
|  |  |  | Female | 7.000 | 0.7656 |
| 3D | SBA vs.  SBA + racemic 3-carene | 10% | Male | 0.000 | >0.9999 |
|  |  |  | Female | 0.000 | >0.9999 |
|  |  | 1% | Male | 10.00 | 0.4062 |
|  |  |  | Female | 6.000 | 0.7656 |
|  |  | 0.1% | Male | 12.00 | 0.2500 |
|  |  |  | Female | -11.00 | 0.2500 |

** Non-significant after Bonferroni correction for multiple comparisons (six per compound).*

**Table S5:** Statistics from Mixed-effects model fitted by REML shown in Figure 4. Significant p-values (p < 0.05) indicated in bold.

| **Figure 4** | **2-Methyl-3-buten-2-ol** | **Verbenone** | **(4*S*)-*cis*-Verbenol** | ***trans*-Verbenol** |
| --- | --- | --- | --- | --- |
| p (day) | **0.0008** | **0.0007** | 0.6092 | **0.0304** |
| F (dfn, dfd) | F (2.210, 40.89) = 0.5319 | F (4, 74) = 5.377 | F (2.210, 40.89) = 0.5319 | F (1.838, 34.00) = 4.005 |
| p (species) | 0.6610 | **0.0275** | **0.0038** | **0.0020** |
| F (dfn, dfd) | F (1, 21) = 10.58 | F (1, 21) = 5.610 | F (1, 21) = 10.58 | F (1, 21) = 12.50 |
| p (day*species) | 0.9521 | 0.0537 | 0.6176 | **0.0015** |
| F (dfn, dfd) | F (4, 74) = 0.6660 | F (4, 74) = 2.447 | F (4, 74) = 0.6660 | F (4, 74) = 4.866 |

**Table S6:** Statistics for results presented in Figure 5. **A)** Unpaired t-test for offspring numbers emerging from Norway spruce (NS) and Scots pine (SP). **B)** General Linear Model (GLM) to assess weight and length of offspring emerging from NS vs SP. **C**) Welch’s test was performed to compare the number of maternal tunnels, GLM was performed to assess length of maternal tunnels in NS and SP.

| **Figure** | **5A** | | |
| --- | --- | --- | --- |
|  | p-value | **0.0156** |  |
|  | df | F_8_ = 5.167 |  |
| **Figure** | **5B** | | |
|  |  | **Weight** | **Length** |
|  | p(sex) | **0.001** | **0.003** |
|  | df | F_1_ = 61.819 | F_1_ = 9.003 |
|  | p(species) | **0.001** | 0.459 |
|  | df | F_1_ = 13.753 | F_1_ = 0.549 |
|  | (sex*species) | 0.836 | 0.848 |
|  | df | F_1_ = 0.043 | F_1_ = 0.037 |
| **Figure** | **5C Number of maternal tunnels** | | |
|  | p-value | **0.0008** |  |
|  | Welch corrected | t=9.449, df=3 |  |
|  | **5C Length of maternal tunnels** | | |
|  | p-value | 0.169 |  |
|  | df | F_1_ = 1.935 |  |

**Method 1**

After volatile collection, the fiber was inserted into a GC coupled to a mass spectrometer (GC-MS; Agilent 8890 GC System, Agilent 5977B GC/MSD) with an inlet temperature of 250 °C. Separation of compounds was performed using an HP-5MS Ultra Inert column (length: 30 m, diameter: 0.25 mm, film thickness: 0.25 µm; J&W Scientific, USA). The stationary phase consisted of 5%Ph-95%Me-Sio. Helium was used as carrier gas. The temperature program started at 45°C held for 1 min, then increasing by 10° C/min up to 250° C, which was held for 5 min with a constant flow rate of 1 mL/min. Quantification of chemicals was done by integrating the peak areas manually. Chemicals were identified by comparing retention times and mass spectra with external standards and a reference library (NIST17).

**Method 2**

Separation of compounds was performed using an HP Chiral 20B column (length: 30 m, diameter: 0.25 mm; film thickness: 0.25 µm; Agilent Technologies) and an HP6890 Series GC System (Agilent Technologies) coupled to an MS (5973 Agilent HP). Helium was used as a carrier gas with splitless injection of 3 µl. The initial temperature was 40° C, then increased by 2° C/min up to 80° C, followed by an increase of 15°C/min up to 230 °C. Chemicals were identified by comparing retention times and mass spectra of with those of external standards and the NIST 17 reference library.
